# Computed tomography‐guided percutaneous microwave ablation: A novel perspective to treat multiple pulmonary ground‐glass opacities

**DOI:** 10.1111/1759-7714.13601

**Published:** 2020-08-03

**Authors:** Baodong Liu, Xin Ye

**Affiliations:** ^1^ Department of Thoracic Surgery Xuanwu Hospital, Capital Medical University Beijing China; ^2^ Department of Oncology The First Affiliated Hospital of Shandong First Medical University & Shandong Provincial Qianfoshan Hospital Jinan China

## Introduction

The increasingly widespread use of computed tomography (CT), high‐resolution CT imaging, and lung cancer screening programs in incidentally detected ground‐glass opacities (GGOs) are more common in routine diagnostic CT imaging, with an estimated detection frequency of 8.72%–27.3% on CT,^1^, ^2^, ^3^ many of which are multiple pulmonary GGOs.

Multiple pulmonary GGOs are defined as more than two GGOs simultaneously found in a single patient. The NELSON study (3392 participants; 7258 nodules) showed that 1746 (51.5%) participants had one nodule, 800 (23.6%) had two nodules, 354 (10.4%) had three nodules, 191 (5.6%) had four nodules, and 301 (8.9%) had more than four nodules.^4^ This suggests that nearly half of the participants had multiple pulmonary GGOs. Moreover, multiple GGOs are an increasingly frequent finding, and 20%–30% of GGOs resected were found to be accompanied by multiple other smaller intrapulmonary GGOs.^5^ Multiple pulmonary nodules are divided into four categories^6^: second primary lung cancer, separate tumor nodules, multiple GGOs, and diffuse pneumonic type.

Multiple pulmonary GGOs are still a “hot potato.” The preferred treatment for GGOs is different and controversial, because multiple GGOs have different imaging characteristics. Several authoritative guidelines do not have a clear opinion on the treatment of multiple GGOs. At present, follow‐up and video‐assisted thoracoscopic surgery (VATS) are the main management modalities for multiple GGOs, which are widely accepted.^7^

## Follow‐up

It is important for physicians to distinguish between transient and persistent GGOs. Transient GGOs disappear during follow‐up. Approximately 40%–50% of GGOs have been reported to disappear after three to four months of follow‐up.^8^, ^9^, ^10^ If the GGOs remain during the follow‐up, the lesions are then classified as persistent GGOs. In general, for GGOs showing no change in size or shape during a 3–4 month follow‐up period, the possibility of lung cancer cannot be completely excluded. Persistent multiple GGOs represent independent histopathologic diagnoses according to size, similar to single GGOs. The treatment strategies and prognosis are not different in terms of the nodule number or size.^11^, ^12^, ^13^ Kim *et al*.^14^ reported that 73 patients underwent pulmonary resection for bronchioloalveolar carcinoma, of whom 23 had multiple GGOs seen on preoperative CT. In the 18 patients who did not have all GGO lesions resected, none of the residual GGO lesions progressed in size or solidity during follow‐up for a median of 40.3 months. Sato *et al*.^15^ reported that 78 patients with multiple GGOs were followed up for a median of 45.5 months, and 37% of the cases had enlargements within the follow‐up period, with most of those appearing within 36 months. Therefore, the recommended optimal observation time for patients with multiple pulmonary GGOs is 36 months. Several studies have reported that after resection of the primary focus, the patients' prognoses will not be affected, regardless of whether other foci continuously grow, a new focus is developed, or the remaining foci are not treated.^16^, ^17^, ^18^ If the secondary focus is a pGGO, and it is impossible to remove all foci due to the limitation of cardiopulmonary functions, it is recommended to follow‐up those patients once every 6–12 months; however, if there is no subsequent change, follow‐up should be done once every two years.

## Video‐assisted thoracoscopic surgery (VATS)

No standard guideline or detailed recommendation for selecting and treating multiple GGOs has been established. The principal treatment strategy for multiple GGOs is complete surgical resection, as long as it is feasible to remove all lesions. However, complete resection for all lesions is difficult for patients with poor cardiopulmonary function, prior pulmonary resection, or those with numerous lesions. Hattori and colleagues believed that in most cases, it is unnecessary to choose complete resection.^19^ Shimada *et al*. and Qu *et al*.^20^, ^21^ suggested that after removing the main lesion, the continuous growth of residual GGO lesions, the presence of a new GGO lesion, or the lack of treatment effect on the remaining GGO lesion will not affect prognosis at all. Making the decision of surgical resection for multiple GGOs is therefore challenging. There is no clear‐cut criterion in choosing an appropriate operative procedure for multiple GGOs, because several clinical factors need to be considered, such as tumor location, size, number, consolidation/tumor ratio, prior pulmonary resection, cardiopulmonary function, and presence of bilateral lesions. The extent of surgical resection is mainly decided by surgeons based on the risk‐and‐benefit balance of surgery, considering the characteristics of the tumor and status of the patient.^22^ It has been suggested that various combinations of sublobar versus lobar resections, of one‐ or two‐stage surgery for bilateral lesions, and of sampling or selected lymph node dissection may be considered. As long as the patient has an adequate pulmonary functional reserve and minimally invasive incision, with no serious complications in the perioperative period, aggressive surgical treatment can achieve favorable long‐term survival. However, standard algorithms do not exist.

## Thermal ablation

Thermal ablation, as a precise minimally invasive technique, has been increasingly used to treat early‐stage lung cancer.^23^, ^24^, ^25^, ^26^ The techniques include radiofrequency ablation (RFA), microwave ablation (MWA), cryoablation, and laser ablation. In recent years, several clinical studies have focused on the outcomes and safety of RFA, MWA, and cryoablation in treating GGO. Kodama *et al*.^27^ reported that lung RFA was performed on 33 patients with 42 lung tumors with ≥50% GGO components. The overall survival (OS) and cancer‐specific survival (CSS) rates were 96.4% and 100% at three years and 96.4% and 100% at five years, respectively. Yang *et al*.^28^ reported that 51 patients with lung adenocarcinoma lesions with GGOs received a total of 52 percutaneous CT‐guided MWA sessions. The median follow‐up period of all the patients was 27.2 months. The rates of three‐year local progression‐free survival (PFS), CSS, and OS were 98%, 100%, and 96%, respectively. The technical success rate was 100%, without MWA procedure‐related death. One of the most common complications for MWA was pneumothorax. Liu *et al*.^29^ retrospectively analyzed the preliminary results of cryoablation of 19 GGNs in 14 patients, and all nodules were completely ablated within the 24 month median follow‐up period. The technical success rate was 100%, without cryoablation procedure‐related death. These results suggest that CT‐guided percutaneous thermal ablation techniques are feasible, safe, and effective therapeutic approaches for treating pulmonary GGO.

At present, the greatest challenge is how to deal with multiple pulmonary GGOs in clinical practice. A study has been published on microwave ablation for multiple pulmonary GGOs, which has attracted much attention. Huang and his colleagues^30^ reported that 33 patients with 103 pulmonary GGOs underwent a total of 66 percutaneous CT‐guided MWA sessions. The median follow‐up period of all patients was 18.1 months. The rates of three‐year local PFS and OS were 100% and 100%, respectively. The technical success rate was 100%, without MWA procedure‐related death. Major complications included pneumothorax (16.7%), pneumonia (4.5%), pleural effusion (3.0%), and nerve injury (1.5%), which were well‐controlled by appropriate treatment modalities. Minor complications included pneumothorax (57.6%), pleural effusion (65.2%), hemoptysis (19.7%), subcutaneous emphysema (4/66, 6.1%), and hemothorax (3.0%). The results are encouraging and may represent a solution for multiple pulmonary GGOs, as well as an alternative treatment for GGOs. This is a very important article and a great contribution for multiple pulmonary GGO treatment.

Certainly, there were several limitations in this study. The main limitations were its retrospective nature, single‐institution study, relatively short follow‐up duration after addressing GGO lesions, as well as the small sample size. Additionally, GGO lesion biopsies were not performed on all lesions in this study, which means that ablation may not be necessary for some lesions. This study was not designed to compare the outcomes of MWA with those of other therapies such as surgery, or RFA. Therefore, a prospective, multicenter, randomized, and controlled study is required to clarify the safety and the effectiveness of MWA in treating multiple pulmonary GGOs.

In the near future, we believe that thermal ablation will definitely become a novel therapy for multiple pulmonary GGOs^7^, ^31^

## Disclosure

No authors report any conflict of interest.
